# Safety of Electrotherapy Treatment in Patients with Knee Osteoarthritis and Cardiac Diseases

**DOI:** 10.3390/life12111690

**Published:** 2022-10-24

**Authors:** Laszlo Irsay, Rodica Ana Ungur, Ileana Monica Borda, Irina Tica, Mădălina Gabriela Iliescu, Alina Deniza Ciubean, Theodor Popa, Delia Cinteza, Florina Ligia Popa, Cosmina Ioana Bondor, Viorela Mihaela Ciortea

**Affiliations:** 1 Department of Rehabilitation Medicine, University of Medicine and Pharmacy, “Iuliu Hatieganu”, 8 Victor Babes Street, 400012 Cluj-Napoca, Romania; 2 Faculty of Medicine, “Ovidius” University of Constanta, 1 University Alley, Campus—Corp B, 900470 Constanta, Romania; 3 Department of Rehabilitation Medicine, Clinical Rehabilitation Hospital, 46-50 Viilor Street, 400337 Cluj-Napoca, Romania; 4 9th Department—Physical Medicine and Rehabilitation, “Carol Davila” University of Medicine and Pharmacy, 37 Dionisie Lupu Street, 020021 Bucharest, Romania; 5 Physical Medicine and Rehabilitation Department, Faculty of Medicine, “Lucian Blaga” University of Sibiu, Victoriei Blvd., 550024 Sibiu, Romania; 6 Academic Emergency Hospital of Sibiu, Coposu Blvd., 550245 Sibiu, Romania; 7 Department of Medical Informatics and Biostatistics, University of Medicine and Pharmacy, “Iuliu Hatieganu”, 6 Pasteur Street, 400349 Cluj-Napoca, Romania

**Keywords:** electrotherapy, contraindications, cardiac arrhythmia, ischemic heart disease, knee osteoarthritis

## Abstract

OBJECTIVE: To assess the safety of electrotherapy applied in the knee area in patients with known atrial arrhythmias or ischemic heart disease, as it is not known whether this treatment induces or aggravates arrhythmias during or immediately after therapy. MATERIAL AND METHODS: The analytical and transversal study involved 46 patients with degenerative knee osteoarthritis (OA), with or without cardiac diseases, from the Clinical Rehabilitation Hospital inpatient center, Cluj-Napoca, Romania. All patients underwent a 10-day physical therapy program for knee OA (electrotherapy, massage and kinesiotherapy). Heart rate and the total number of ventricular and supraventricular extrasystoles were evaluated before and after treatment, by 24 h Holter ECG monitoring. RESULTS: There was no significant increase in heart rate or in the number of ventricular or supraventricular extrasystoles before or after electrotherapy treatment, regardless of the positive or negative history of arrhythmia or ischemic heart disease (all *p* > 0.05). Mean values during day 1 were: 35.15 (95% CI [9.60–60.75]) for ventricular ones extrasystoles and 91.7 (95% CI [51.69–131.7]) for supraventricular ones, which during day 2 were 38.09 (95% CI [3.68–72.50]), 110.48 (95% CI [48.59–172.36]), respectively. CONCLUSION: One of the most important things to consider when dealing with an OA patient is that they are most likely older than 65 years, which increases the chance of having a cardiac disease. This raises the need for viable interventions regarding the management of this disease in patients that probably have multiple comorbidities, and where pharmacological and surgical management are not possible, limited or have multiple side effects. Electrotherapy used for treating knee OA did not cause a significant increase in heart rate or number of ventricular and supraventricular extrasystoles in this category of patients.

## 1. Introduction

Electrotherapy (ET) is defined as the use of electrical energy as a medical treatment and is one of the fundamental elements of physiotherapy (PT) practice [1]. ET is often poorly understood, sometimes misused and the topic of multiple debates that have occurred regarding its efficiency and safety. Regardless of its controversial status, it has various known effects and indications: muscle spasm relaxation, muscle stimulation, muscular atrophy, increase in blood circulation, maintaining and increasing range of motion, decrease of posttraumatic acute and chronic pain, etc. [2].

The application of electrotherapy for the treatment of various musculoskeletal pathologies is frequent in the population of postmenopausal women not only for osteoarthritis (OA), but osteoporosis, too. In our region, the burden of postmenopausal osteoporosis and its complications is increasing and the cardiac safety of the therapy is an additional concern regarding this population OA [3,4].

ET is often used to manage symptoms of chronic diseases, one of which is osteoarthritis. It is considered a relatively inexpensive and noninvasive, short-term treatment option, and it is recommended in evidence-based clinical guidelines [5,6,7,8]. The most widely used and studied PT and ET treatments for knee OA seem to be Transcutaneous Electrical Nerve Stimulation (TENS), ultrasound, interferential and galvanic current, laser therapy, neuromuscular electrical stimulation and shortwave diathermy [9,10,11,12,13,14,15,16,17]. Unfortunately, not all patients can benefit from this form of therapy. There are certain contraindications and/or precautions when applying this type of treatment in patients with or without cardiac diseases. Even though ET is widely used, research is scarce regarding its effects in patients with associated arrhythmias or ischemic heart disease, and it is not known whether ET itself induces or aggravates certain arrhythmias during or immediately after therapy. The current treatment recommendations include relative contraindications or precautions in patients with different cardiac diseases, but without giving any details or providing information to support this statement. In many situations, the relative contraindication is given by the lack of studies, and not for the known cardiac adverse effects of ET [18]. It is difficult to identify if the electrical modifications that occur in patients treated by ET modalities are related to it, or their appearance is spontaneous with no causal connection. In a study conducted on healthy volunteers, 24 h Holter ECG monitoring showed that 60.8% had premature atrial contractions (PAC), 2.2% had supraventricular tachycardia, 0.1% had atrial fibrillation (AF) and 2.4% had type II atrioventricular block [19]. Being able to recognize the preceding conditions that could determine AF can lead to early diagnosis and prevention/decrease in morbidity and mortality. Recent studies show that having more than 100 PAC a day is a major risk factor for developing AF [20]. In individuals aged 50 or more, PAC occur in correlation with age, height, other cardiac diseases, physical activity and serum levels of cholesterol and natriuretic peptides [21]. Monitoring extrasystoles is difficult, due to their variation dependence on the circadian rhythm and blood pressure. High blood pressure favors the occurrence of ectopic ventricular beats, and diuretic treatment decreases their frequency [22]. The purpose of the present study is to evaluate whether ET is a valid influence in inducing extrasystolic arrhythmia in patients treated for knee OA, without cardiac pathology, as well as in those with known arrhythmias or ischemic heart disease.

## 2. Materials and Methods

An analytical and transversal study was carried out between July 2021 and November 2021. The first patient was recruited in July 2021, and the last patient finished the follow-up in November 2021. The study included a total of 46 patients previously diagnosed with degenerative knee OA. All the patients included in the study were recruited during their inpatient visit to the Clinical Rehabilitation Hospital in Cluj-Napoca, Romania. The inclusion criteria were as follows: clinical diagnosis and imaging diagnosis of knee OA based on ACR 2000 diagnostic criteria. The exclusion criteria were as follows: known severe cardiac arrhythmias (AF, atrial flutter), congestive heart failure class NYHA II, III or IV and any other general contraindications for PT (infections, psychiatric disorders etc.). Patients with myocardial ischemia or PAC were allowed to enter the study, since we considered it useful to observe whether during ET treatment the cardiac diseases were aggravated or other complications occur.

**Figure 1:**
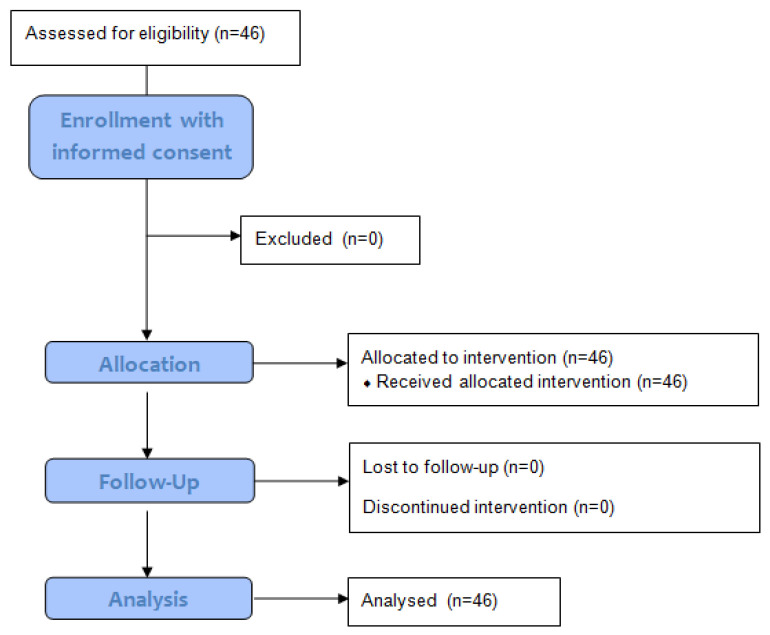
Flowchart of our study.

Each patient was clinically evaluated and was furthermore prescribed a PT program (ET, massage and exercise therapy) by their doctors, based on their clinical judgment and with respect to the patient’s contraindications. The investigators in this study did not interfere with the choice of PT treatment, which is why we did not consider registering the study before starting enrollment. They were monitored by 24 h Holter ECG for 2 separate days at the beginning of treatment, before applying the PT methods (day 1), and after completing a 10-day PT program (day 2). The results recorded by the Holter were interpreted by a fellow cardiologist. The data obtained was: heart rate (minimum, average and maximum) and number of extrasystoles (supraventricular, ventricular and total). PT modalities prescribed for each patient after clinical evaluation included different types of ET applied in the knee area, as in low frequency currents (galvanic or TENS), medium frequency currents (interferential) and high frequency currents (short wave diathermy), which were combined with an individualized exercise program and massage therapy. The program was performed on a daily basis for 10 days. The aim of the PT program was to decrease pain and increase range of motion in the affected joints without leading to any increase in cardiac frequencies. No hydrotherapy was applied, given that that the Holter ECG was not waterproof. No technical incidents were reported during the study. No alternate positioning of the patient wearing a Holter ECG for ET procedures was necessary, as the region of interest was the knee. No change in current drug therapy was allowed during the whole duration of the study. The flowchart of the study can be seen in Figure 1.

Statistical analysis has been performed using SPSS. The distribution normality of the data has been tested using Shapiro-Wilk test. The Wilcoxon Paired Signed-Rank test has been used to verify whether there is a statistically significant change between the recorded data of the two days. A *p*-value < 0.05 was considered as statistically significant.

The study was approved by the Ethics Committee of the University of Medicine and Pharmacy “Iuliu Hatieganu” Cluj-Napoca. The participants were informed of the characteristics of the study, and all of them gave signed informed consent prior to inclusion. This study was conducted in accordance with the 2008 Declaration of Helsinki. The authors confirm that all ongoing and related trials for this drug/intervention are registered.

## 3. Results

A total of 46 patients met the inclusion criteria, of which 33 were female and 13 were male. The main characteristics of the patients included in the study are shown in Table 1.

The mean age was 64.7 years (CI 95% [59.25–66.05]), and the median age was 66 years. Of the 46 patients, 26 did not have any ECG modifications and did not have a cardiac disease, 20 were known with PAC and 19 had been previously diagnosed with ischemic heart disease.

Statistical analysis of the 46 patients did not reveal significant modifications regarding the incidence of ventricular or supraventricular extrasystoles (Table 2).

The mean value for ventricular extrasystoles during day 1 was 35.15% (95% CI [9.60–60.75]), with a median of 3, and during day 2 was 38.09% (95% CI [3.68–72.50]), with a median of 3.5. As for supraventricular extrasystoles, the mean value was 91.7 (95% CI [51.69–131.7]), with a median of 36 during day 1, and 110.48 (95% CI [48.59–172.36]), with a median of 29.5 during day 2, respectively.

No significant modifications occurred in minimal, average or maximum heart rate per minute before or after the application of the PT methods, as shown in Table 3 (all *p* > 0.05).

When dividing patients into groups according to their positive or negative history of extrasystolic arrhythmia or ischemic heart disease, no statistical significance was found for any of the correlations (all *p* > 0.05). The data obtained can be seen in Table 4.

## 4. Discussion

To our knowledge, this is the first study to assess whether an ET program for knee OA in patients with or without known extrasystolic arrhythmias or ischemic heart disease can alter the pre-known condition or can induce ECG modifications. As is well known, OA is a chronic and degenerative joint disease and is considered one of the most common musculoskeletal disorders [23]. It is estimated that more than 85% of patients present with radiographic evidences of OA after 65 years [24]. The main clinical symptoms reported by patients with knee OA are: pain, articular stiffness, joint deformities, loss of range of motion, muscle weakness and limitations in physical activity [25]. For these reasons, several pharmacological, non-pharmacological and surgical interventions have been implemented to deal with these patients. PT is one of the main non-pharmacological strategies that plays a fundamental role in patient management, of which kinesiotherapy (therapeutic exercises) and ET modalities are the most widely used [26].

The present study included a total of 46 patients previously diagnosed with degenerative knee OA, who were prescribed a PT program to decrease pain and increase range of motion in the affected joint. The ET consisted of low, medium or high frequency currents, applied in the knee area for 10 consecutive days. Results obtained by Holter ECG showed that the use of ET for treating knee OA did not cause a significant increase either in the number of ventricular or supraventricular extrasystoles or in minimum, average or maximum heart rate, regardless of their positive or negative history of arrhythmia or ischemic heart disease.

One of the most important things to consider when dealing with an OA patient is that most patients are likely older than 65 years, which increases the chance of having a cardiac disease. This raises the need for viable interventions regarding the management of this disease in patients that probably have multiple comorbidities, and where pharmacological and surgical management are not possible, limited or have multiple side effects. Pain is one of the most common complaints and disabling symptoms in an OA population, and it is responsible for a low functional capacity and quality of life. All ET modalities can be used individually or combined to pursue pain, swelling and inflammation reduction and increase or maintain muscle strength, as reported in multiple studies [9,10,11,12,13,14,15,16,17].

The decision to use a form of ET for certain pathologies is based on the knowledge of their physical and physiological properties. Due to the fact that information on how to deal with specific scenarios is often lacking, physical medicine and rehabilitation doctors tend to act conservatively when prescribing treatments in certain situations. Since research is sometimes unavailable due to ethical restrictions (e.g., pregnancy), the conservative approach is therefore acceptable. But most of the time, cited contraindications are often based more on a common sense rather than an evidence-based approach in clinical practice [27]. The current absolute contraindications for ET in patients with cardiac diseases include those with cardiac pacemakers, cardiac arrhythmias (AF or atrial flutter) and congestive heart failure. But precaution or no treatment is advised in patients with other types of cardiac arrhythmias (uncontrolled, causing symptoms or hemodynamic compromise), unstable angina pectoris, resting ECG abnormalities (recent ST displacement or elevation), tachy- or bradyarrhythmias and atrioventricular blocks (high degree) [28]. Knowledge of the contraindications to ET modalities is essential for safe and effective treatment. All forms of ET carry an inherent risk to the patient if not prescribed or applied correctly. A small number of research studies have investigated the issue of contraindications to various ET modalities, but these vary in quality. Much of the literature used is based on single case studies, a lower quality of evidence, but nonetheless useful in considering different clinical scenarios when planning patient care [29,30]. Also, limited information is available from manufacturers.

It is a known fact that arrythmias in healthy subjects are relatively frequent. A recent study involving 1273 subjects monitored by Holter ECG for 24 h, aged between 18 to 65 years, revealed the presence of PAC in 60.8% and AF in 0.1% of the subjects [19]. Also, long term and sustained sport activities in young and middle-aged men increase the risk of developing AF over time, whereas in older men, moderate physical activity decreases the risk [31]. In women, there was no significant difference regarding the occurrence of AF, regardless of their physical activity level [32].

In the current study, the intensity of the exercises were low to moderate, which is why we can safely assume that the PT program prescribed for the knee OA did not increase the risk of developing supraventricular or ventricular arrythmias.

The main limitations of the study are the low number of participants and lack of variety in cardiovascular pathology. Also, the effects of PT procedures were not analyzed separately, but cumulatively, which is closer to the clinical reality, as patients undergo multiple types of PT procedures altogether. On the other hand, we believe it is safe to assume that if multiple procedures applied to the same patient did not produce alterations on the ECG, the individual application of one procedure will probably not have these undesired effects also [33].

## 5. Conclusions

Electrotherapy methods applied in the knee area did not cause an increase in the number of ventricular or supraventricular extrasystoles. The positive history of extrasystolic arrhythmia or stable ischemic heart disease should not be considered a contraindication for peripheral application of ET.

The present analysis suggests a need for further research, in order to potentially reassess the current cardiac contraindications for ET treatment and to bring more evidence regarding the safety of ET application in patients with different cardiac diseases.

## Figures and Tables

**Table 1:** Clinical characteristics of the patients included in the study.

n = 46		
Age (years)	Mean	Median
	62.7 (95% CI [59.25–66.05]	66
Sex	Female	Male
	n = 33	n = 13
Ischemic cardiac disease	Present	Not present
	n = 19	n = 27
Extrasystolic Arrhythmia	Present	Not present
	n = 20	n = 26

**Table 2:** Analysis of the number of ventricular, supraventricular and total number of extrasystoles in the study population before and after ET treatment.

Extrasystoles (n = 46)		Mean (95% CI)	Median	*p*-Value *
Number of ventricular extraystoles/24 h	Day 1	35.17 [9.6–60.75]	3	0.974
	Day 2	38.9 [3.68–72.5]	3.5	
Number of supraventricular extrasystoles/24 h	Day 1	91.7 [51.69–131.7]	36	0.642
	Day 2	110.48 [48.59–172.36]	29.5	
Number of total extrasystoles/24 h	Day 1	126.87 [76.63–177.11]	55	0.407
	Day 2	148.57 [75.57–221.56]	41	

* Wilcoxon signed rank test.

**Table 3:** Analysis of heart rate/minute before and after ET treatment.

Heart Rate/Minute (n = 46)		Median	*p*-Value *
Minimal	Day 1	49 [44–53.5]	0.969
	Day 2	50 [45–53.75]	
Average	Day 1	67 [59–75]	0.599
	Day 2	68 [61.25–72]	
Maximal	Day 1	114 [100.5–136]	0.729
	Day 2	115 [101.75]	

* Wilcoxon signed rank test.

**Table 4:** Comparison between patients with and without known extrasystolic arrhythmias and with or without ischemic heart disease.

n = 46		History of Extrasystolic Arrhythmias			History of Ischemic Heart Disease		
		Negative (n = 26)			Negative (n = 27)		
		Mean (95% CI)	Median	*p*-Value	Mean (95% CI)	Median	*p*-Value
Number of ventricular extrasystoles/24 h	Day 1	26.46 [−4.82–57.74]	1	0.395	34.3 [−1.18–69.78]	3	0.576
	Day 2	13.58 [0.49–26.66]	1.5		44.52 [−11.69–100.73]	3	
Number of supraventricular extrasystoles/24 h	Day 1	51.35 [23.87–78.83]	30	0.989	63.41 [22.01–104.8]	12	0.731
	Day 2	45.31 [21.12–69.5]	23.5		67.33 [1.68–132.99]	29	
Number of total extrasystoles/24 h	Day 1	77.81 [32.91–122.7]	36	0.426	97.7 [39.19–156.22]	36	0.475
	Day 2	58.88 [28.78–88.99]	27.5		111.85 [21.45–202.25]	37	
		**Positive (n = 20)**			**Positive (n = 19)**		
Number of ventricular extrasystoles/24 h	Day 1	46.5 [1.05–91.95]	6	0.393	36.42 [−3.66–76.5]	5	0.489
	Day 2	69.95 [−9.09–148.99]	20.5		28.95 [−2.46–60.36]	8	
Number of supraventricular extrasystoles/24 h	Day 1	144.15 [60.73–227.57]	45.5	0.528	131.89 [53.21–210.58]	56	0.546
	Day 2	195.2 [58.94–331.46]	44		171.79 [51.76–291.82]	77	
Number of total extrasystoles/24 h	Day 1	190.65 [92.54–288.76]	74	0.061	168.32 [75.79–260.84]	62	0.494
	Day 2	265.15 [109.6–420.7]	88		200.74 [72.57–328.91]	85	

## Data Availability

All data generated or analyzed during this study are included in this published article.

## Abbreviations

AF: atrial fibrillation; ET: electrotherapy; PAC: premature atrial contractions; PT: physiotherapy; OA: osteoarthritis; TENS: Transcutaneous Electrical Nerve Stimulation.
